# Stroke Presenting as a Complication of Sarcoidosis in an Otherwise Asymptomatic Patient

**DOI:** 10.7759/cureus.2362

**Published:** 2018-03-26

**Authors:** Muhamad Memon, Muhammed AlHazza, Humariya Heena

**Affiliations:** 1 National Neurosciences Institute, King Fahad Medical City, Riyadh, Saudi Arabia, Riyadh, SAU; 2 Research Center, King Fahad Medical City, Riyadh, Saudi Arabia

**Keywords:** biatrial enlargement, restrictive cardiomyopathy, lt. middle cerebral artery, implantable cardioverter-defibrillator device, bihilar lymphadenopathy

## Abstract

A stroke occurring in young patients in the absence of common risk factors needs a thorough investigation of the underlying cause to prevent its recurrence. Herein, we discuss a case of stroke with rare etiology in a 28-year-old male presenting within 30 minutes of speech difficulty and right-sided weakness. The initial triage workup showed an abnormal configuration of the P wave in the 12 lead echocardiograph (ECG) and his chest x-ray (CXR) showed mediastinal widening. His echocardiogram and chest computed tomography (CT) confirmed bilateral enlargement with restrictive cardiomyopathy and mediastinal lymphadenopathy, raising a suspicion of sarcoidosis. A cardiac positron emission tomography (PET) scan confirmed the diagnosis by showing a non-caseating granuloma. The patient was put on intravenous (IV) tissue plasminogen activator (TPA) and his National Institute of Health Stroke Scale (NIHSS) came down from 14 on admission to zero within 48 hours. Cardiac involvement in sarcoidosis is not uncommon but it presenting as stroke is extremely rare. For a young, previously healthy patient presenting as a stroke without risk factors, sarcoidosis should be considered as a differential diagnosis.

## Introduction

Sarcoidosis is a multisystem inflammatory disease that involves small vessels [1]. Cardiac and neurological involvement is rare in sarcoidosis [2]. Only 5% of patients present with neurosarcoidosis [2]. Patients with neurosarcoidosis can present with various presentations, such as craniopathies, meningoencephalitis, peripheral nerve involvement, and psychiatric symptoms [3]. It affects women more than men [4]. The prevalence of sarcoidosis has increased in the last 15 years, especially in the USA and occurs roughly in 50 out of 100,000 people [4]. Cardiac involvement in sarcoidosis is in the order of 20%-30%, out of which only 5% develop primary cardiac sarcoidosis, which is a leading cause of death in patients with sarcoidosis, with a mortality rate varying from 25% to 85% [5-6]. However, the data for sarcoidosis prevalence and complications in the Kingdom of Saudi Arabia (KSA) is limited. A few studies done in Saudi Arabia emphasize that the clinical presentation of these patients are similar to the western pattern of the disease but cardiac involvement is rare [7]. Due to the diverse nature of the disease and the involvement of small vessels, sarcoidosis rarely presents as a large artery stroke [7]. Since stroke is a disabling disease, therefore, correct assessment and a management plan are vital to address the recurrence of stroke.

Herein, we present a case of undiagnosed sarcoidosis with cardiac involvement presenting as a large artery stroke, reinforcing the importance of keeping this differential diagnosis in mind while evaluating young stroke cases.

## Case presentation

**Figure 1:**
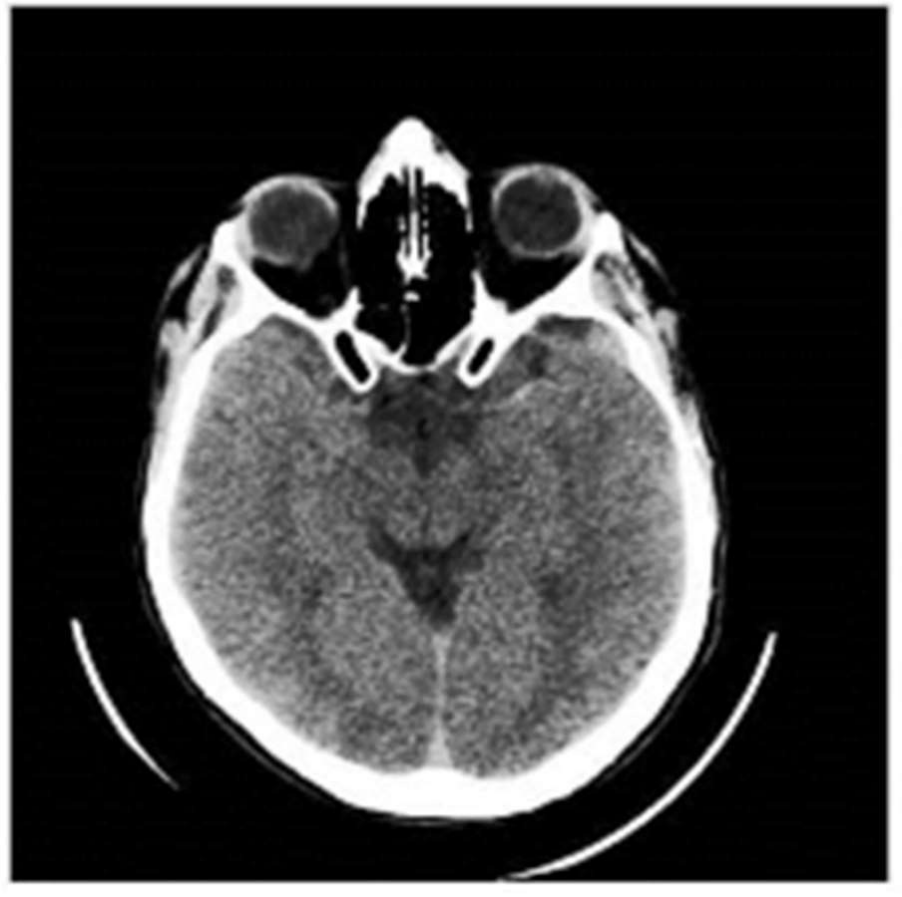
CT head showing a left hyperdense middle cerebral artery sign Computed tomography (CT) head with a left hyperdense middle cerebral artery sign

**Figure 2:**
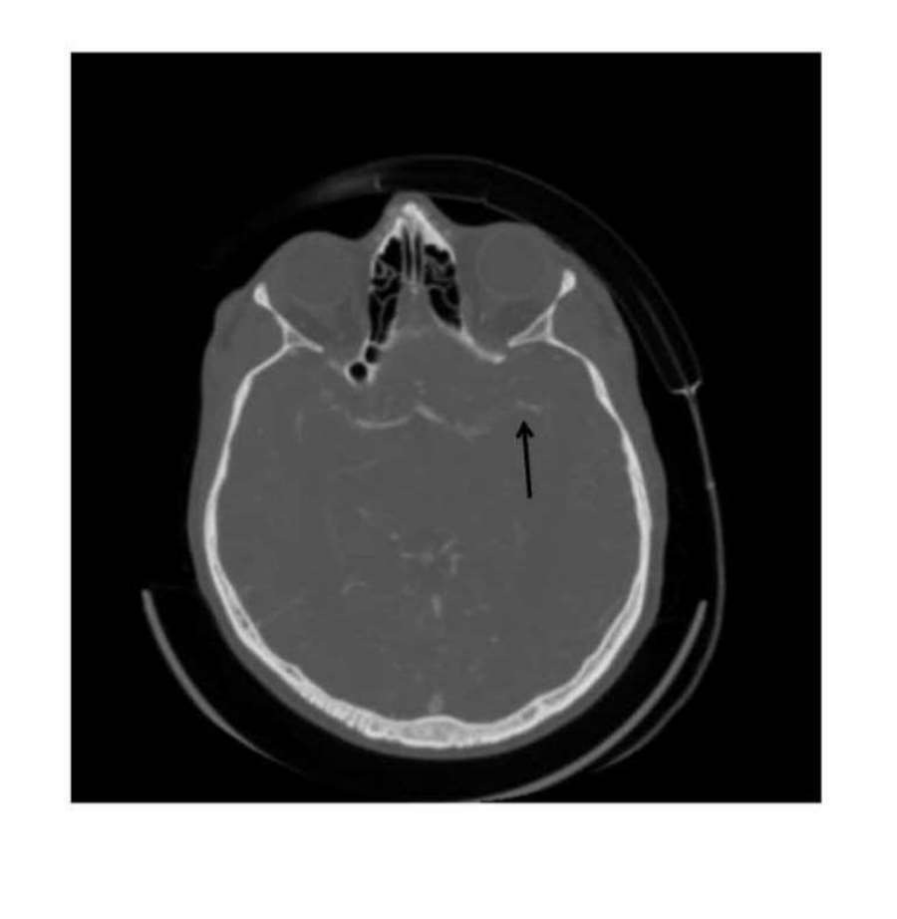
CT angiogram showing the cut-off of blood supply at the M2 part of the left middle cerebral artery Computed tomography angiogram (CT) showing the cut-off of blood supply at the M2 part of the left middle cerebral artery

A 28-year-old male presented to the King Fahad Medical City (KFMC) emergency department (ED) within one hour of the onset of speech difficulty and right-sided weakness while he was driving his kids to school in the morning. He was a nonsmoker with a healthy lifestyle and normal body mass index (BMI) and apart from tonsillectomy as a child, he did not have any significant medical or surgical history and was not on any medications. In the triage of the emergency room, the stroke code was announced where he was rapidly triaged, assessed by the stroke team, and rushed to radiology for computed tomography (CT) and computed tomographical angiogram (CTA) (Figure 1 and Figure 2, respectively).

On admission to ED, his blood pressure, pulse, and random blood sugar were within normal limits, and his National Institute of Health Stroke Scale (NIHSS) was 14, which pointed toward severe stroke.

His CT scan showed left hyperdense middle cerebral artery sign with CT angiogram (CTA) showing a cut-off of the blood supply at the M2 part of the left middle cerebral artery.

**Figure 3:**
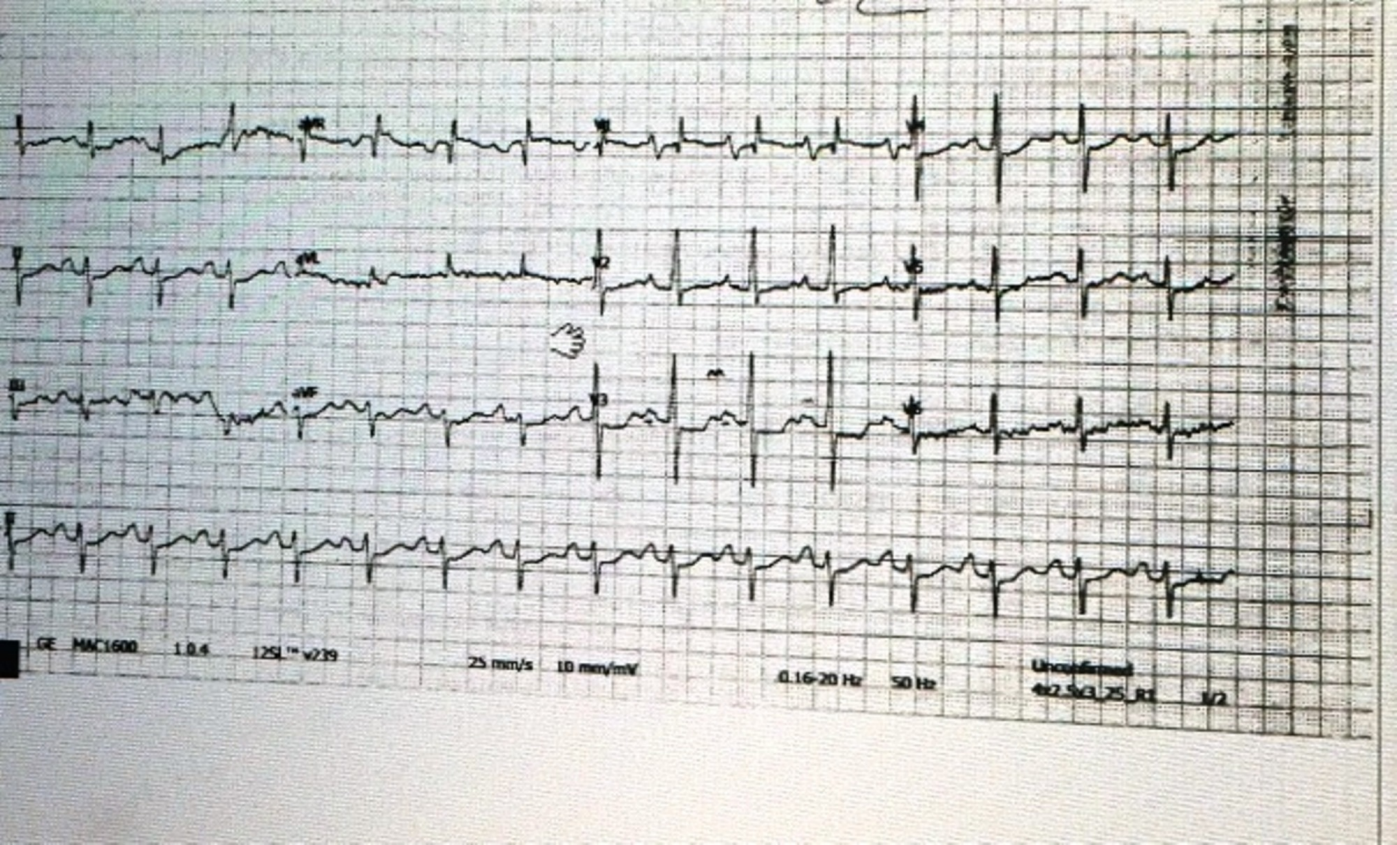
Twelve lead ECG showing septal hypertrophy V2, 3, 4, bifid P wave in lead II, V3 Twelve lead echocardiogram (ECG) showing septal hypertrophy V2, 3, 4, bifid P wave in lead II, V3

His baseline ECG showed septal hypertrophy and biatrial hypertrophy (Figure 3).

**Figure 4:**
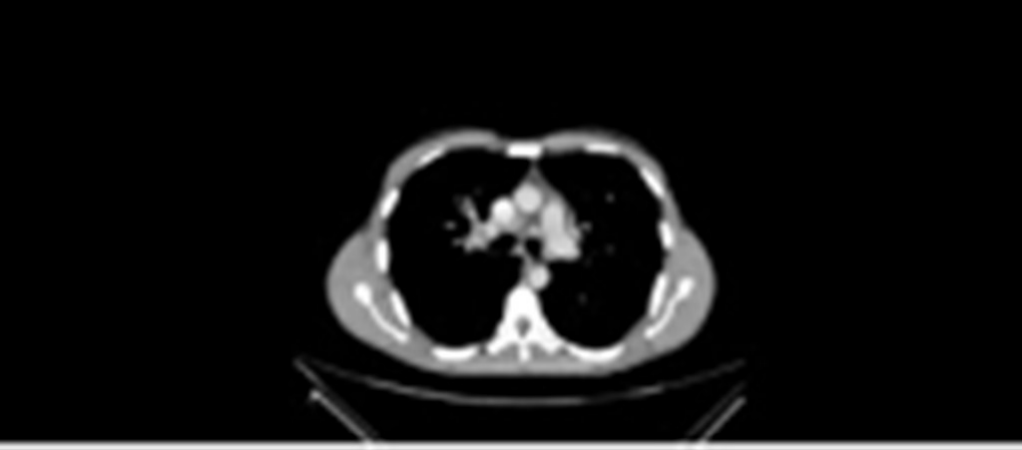
CT chest showing bihilar lymphadenopathy Computed tomography (CT) chest showing bihilar lymphadenopathy

He was treated with IV tissue plasminogen activator (TPA) within 56 minutes of presentation to the ED and made a remarkable recovery after IV TPA. Within 24 hours, his NIHSS scale improved from 14 to 2, and he was able to perform most of his daily activities the very next day. His ECG was strongly suggestive of biatrial enlargements with his stroke mainly cardioembolic in origin. Echo showed restrictive cardiomyopathy, and further imaging was advised. CT chest (Figure 4) showed hilar and mediastinal lymphadenopathy.

There was no significant finding in CT abdomen/pelvis. His polymerase chain reaction (PCR)/3+ sputum samples for Mycobacterium tuberculosis were all negative. We suspected that he was suffering from the rare heart condition of cardiac sarcoidosis due to which he developed restrictive cardiomyopathy causing a paroxysmal irregular rhythm of the heart, leading to a cardioembolic stroke. He underwent mediastinal lymph node biopsy and a cardiac positron emission tomography (PET) scan, which showed a non-caseating granuloma. The PET scan confirmed the diagnosis of cardiac involvement typical of sarcoidosis. The thrombophilia screen was negative. Serum angiotensin-converting enzyme (ACE) levels were not obtained, as this facility was not available in our hospital.

Treatment and follow-up

He was started on high-dose steroids, referred to rheumatology and cardiology, and started on steroid-sparing immunosuppressive medications because he could not tolerate the steroids.On follow-up, he was recovering well, asymptomatic, with no neurological deficit, and his NIHSS came down to zero and his modified Rankin (mRS) score was zero as well. He responded very well to steroid-sparing immunosuppressive medications and is still under rheumatology follow-up, receiving infliximab injections, with his recent PET scan showing marked improvement. 

## Discussion

Sarcoidosis is a chronic granulomatous condition that affects almost every organ and causes inflammatory changes in small vessels. Mortality is significantly high if it affects the heart. The usual presentation in sarcoidosis with cardiac involvement is of conduction abnormalities or heart failure secondary to systolic dysfunction and, very rarely, cardioembolic stroke.

Sarcoidosis can affect the heart in many different ways with 32% of patients presenting with conduction abnormalities and 75% of patients developing heart failure [5-6]. Patients with cardiac sarcoidosis (CS) develop restrictive cardiomyopathy initially, causing diastolic dysfunction of the heart, with 65% of patients presenting as sudden cardiac death [5-6]. Some patients may either progress to systolic dysfunction and dilated cardiomyopathy in late stages of the disease or develop features of pulmonary hypertension and cor pulmonale [1]. Stroke is an extremely rare complication of sarcoidosis, with not many cases encountered that are cardioembolic in origin.

We have presented a case of hyperacute stroke with restrictive cardiomyopathy secondary to the extrapulmonary manifestation of sarcoidosis in a previously asymptomatic young adult, which is proven by a lymph node biopsy and PET scan.

We searched PubMed and found only a few cases of sarcoidosis presenting as stroke [7-9]. In each case, patients were known to have systemic sarcoidosis, and their presentation was secondary to other complications of systemic sarcoidosis not cardiac sarcoidosis present with a large artery stroke. Our patient did not have a prior diagnosis of sarcoidosis; he was completely asymptomatic before this event.

In our patient, the neurological deficit was secondary to the cardioembolic source and was fully resolved with IV TPA and he had a complete neurological recovery.

## Conclusions

We have presented a case of a hyperacute stroke secondary to the extrapulmonary manifestation of sarcoidosis in a previously asymptomatic young adult. Our patient had echo findings of restrictive cardiomyopathy and suffered from a cardioembolic stroke rather than presenting with a typical neurosarcoidosis presentation.This emphasizes the importance of keeping stroke as the differential diagnosis in a previously asymptomatic healthy patient achieving complete neurological recovery with appropriate treatment.
